# Frequency of familial hypercholesterolaemia-causing genetic variants in the 100 000 Genomes Project cohort: whole genome sequencing analyses of 77 260 participants

**DOI:** 10.1136/jmg-2025-111201

**Published:** 2026-02-10

**Authors:** Marta Futema, Martin Bird, Ash Haeger, Ellen Pinder, Anthony O’Rourke, Elijah R Behr, Steve E Humphries

**Affiliations:** 1 Cardiovascular and Genomics Research Institute, School of Health & Medical Sciences, City St George’s University of London, London, UK; 2 Institute of Cardiovascular Science, Faculty of Population Health, University College London, London, UK; 3 Clinical Pharmacology and Precision Medicine, William Harvey Research Institute, Queen Mary University of London, London, UK; 4 Oxford Genetic Laboratories, Oxford University Hospitals NHS Foundation Trust, The Churchill Hospital, Oxford, UK

**Keywords:** Genomics, Mutation, Cardiovascular Diseases

## Abstract

**Background:**

Heterozygous Familial Hypercholesterolaemia (HeFH) is caused by pathogenic variants in *LDLR*, *APOB*, *APOE* or *PCSK9*, leading to elevated low-density lipoprotein-cholesterol and increased cardiovascular risk. In the UK, HeFH affects ~1 in 288 individuals. The 100 000 Genomes Project (100KGP) generated whole genome sequencing (WGS) data from >85 000 participants recruited primarily with cancer or rare inherited disorders. We analysed WGS data to assess the prevalence and spectrum of FH-causing variants.

**Methods:**

Variants in *LDLR*, *APOB*, *APOE* and *PCSK9* were extracted from 100KGP WGS data and annotated using expert-reviewed ClinGen curation. Demographic, ancestry and linked health records were incorporated. Analyses were restricted to unrelated individuals.

**Results:**

Among 54 818 unrelated participants, 167 were heterozygote for an FH-causing variant, giving a prevalence of 1:328 (95% CI 1:285 to 1:386). Prevalence was similar across ancestries, including African (1:388) and South Asian (1:276). Variant distribution was: *LDLR* 67%, *APOB* 26.5%, *APOE* 3.5% and *PCSK9* 3%. Two individuals carried two FH variants, consistent with homozygous FH. Among 22 442 genetic relatives, 77 also carried an FH variant. Of all variant carriers, 53% were female, mean age at recruitment was 41.3 years, with 43 younger than 18 years, and 54.3% had documented hypercholesterolaemia.

**Conclusions:**

The prevalence and gene distribution of FH-causing variants in 100KGP are consistent with UK estimates. Differences in variant spectrum across ancestries were observed; however, FH prevalence was similar. Participants who consented to the return of actionable findings were informed, providing direct clinical benefit from genomic research.

**WHAT IS ALREADY KNOWN ON THIS TOPIC**

- Heterozygous Familial Hypercholesterolaemia (HeFH) is a common inherited cause of elevated low-density lipoprotein-cholesterol, affecting around 1 in 288 individuals in the UK. Previous studies have largely relied on clinical cohorts or targeted genetic screening, with limited data on variant distribution across diverse ancestries.

**WHAT THIS STUDY ADDS**

- This large-scale analysis of whole genome sequencing data from the 100 000 Genomes Project confirms the prevalence of FH-causing variants (at around 0.3%), consistent with prior estimates. It demonstrates differences in the spectrum of causal genes across ancestries and identifies affected individuals, including children, who may benefit from early diagnosis and intervention.

**HOW THIS STUDY MIGHT AFFECT RESEARCH, PRACTICE OR POLICY**

- These findings support the value of population-scale genomics in accurately determining disease prevalence, improving detection of at-risk individuals and informing cascade screening strategies. Incorporating ancestry-specific insights may refine genetic testing approaches, and return of actionable findings illustrates the potential of genomic research to deliver immediate health benefits.

## Introduction

 The 100 000 Genomes Project (100KGP) was launched in the UK in 2013 to investigate the role of whole genome sequencing (WGS) in the National Healthcare Service (NHS) setting to provide molecular diagnosis to participants with rare diseases and cancers.^1^ While ~17 000 of recruits had cancer (with WGS performed on both somatic and tumour samples), there were over 70 000 samples from individuals, or families where a proband had an undiagnosed but likely genetic disorder. The project has already resulted in the identification of many novel genetic causes and led to the molecular diagnosis of the children, allowing better management of the affected individuals and counselling of their relatives.^1^ Eligibility to take part in the 100KGP included individuals with a clinical diagnosis of Familial Hypercholesterolaemia (FH). This is an autosomal dominant disorder, characterised by having, from birth, elevated concentrations of the atherogenic low-density lipoprotein-cholesterol (LDL-C) particle. This results in the early development of atherosclerosis and a high risk of premature coronary artery disease (CAD), including myocardial infarction. Regardless of the LDL-C concentration, individuals with a pathogenic FH variant are at higher risk of CAD, when compared with LDL-C-matched subjects with no pathogenic FH variants.^2^ Individuals with FH, once identified, can be given healthy lifestyle advice and offered treatment with lipid-lowering therapy (LLT) such as statins or inhibitors of PCSK9.^3^ Such treatment has been shown to result in a considerable reduction in the risk of developing CAD and to reduce CAD morbidity and mortality.^4^

FH is caused by having a pathogenic variant in any of four genes involved in the clearance of LDL-C, namely the *LDLR*, *APOB*, *APOE* and *PCSK9* genes. We have previously shown that in the 536 FH individuals recruited into the 100KGP, an FH-causing variant was found in 17% with ~40% having a polygenic aetiology for their hypercholesterolaemia and 14% having genetically determined high concentrations of a related atherogenic lipoprotein called Lp(a), with the remainder having an unknown cause for their FH phenotype.^5^ However, one of the issues with all such genetic testing is to establish whether an identified variant is pathogenic or not. The American College of Medical Genetics and Genomics (ACMG) has developed criteria to distinguish pathogenic (P) and likely pathogenic (LP) variants from Variants of Uncertain Significance (VUS) or benign (B) or likely benign (LB).^6^ For variants in *LDLR,* these criteria have been further adapted by the FH ClinGen consortium.^7^ For FH genetic testing, the frequency of VUS in a recent UK-wide survey of over 9500 patient samples in the UK Genomic Laboratory Hubs was ~2%.^8^

In addition to receiving their main findings for their known cancer or rare disease, 100KGP participants also had the option to consent to receive additional findings (AFs), for diseases having clinically useful actions available to reduce future risk.^9^ AFs for adults were pathogenic variants in 13 genes associated with an increased risk of some cancers (*MLH1, MSH2, MSH6, MUTYH, APC, BRCA1, BRCA2, VHL, MEN1, RET*) or FH (*LDLR, APOB, PCSK9*). AFs for children (*MUTYH, APC, VHL, MEN1, RET, LDLR, APOB, PCSK9*) excluded genes for adult-onset conditions.

Surveys and interviews conducted with participants who received an AF result for risk of cancer or FH have reported that such results were generally seen as useful and would influence health management.^10^ Although those receiving a cancer AF were often initially ‘shocked’ and ‘anxious’ and found telling family members difficult, no participants with FH AFs described feeling distressed on learning their result, with many reporting that the FH result was not a surprise as they or a family member had high cholesterol or had previously received an FH diagnosis via another pathway.

In a recent paper,^11^ the potential cost benefit of AFs for a number of disorders (including FH) was examined in a subgroup of ~17 000 100KGP participants, suggesting that returning these AFs would be clinically useful and cost saving. A more detailed analysis suggests that providing FH AFs is likely to be below the NICE threshold for being cost effective, particularly if costs for bioinformatic analysis could be reduced.^12^ Here, we present an analysis of the WGS data on the entire 100KGP cohort, to document the frequency and spectrum of FH-causing variants in this cohort in subjects of different ancestry.

## Methods

### 100KGP cohort

The 100KGP^13^ was approved by East of England–Cambridge Central Research Ethics Committee ref:20/EE/0035. Only participants who provided written informed consent for their data to be used for research were included in the analyses. The current project (RR127) has been approved by the Genomics England Clinical Interpretation Partnership cardiovascular domain committee.

The cohort, comprising 77 260 participants included in the aggregated variant calls dataset (AggV2 (https://re-docs.genomicsengland.co.uk/aggv2/)), generated from Genomics England data release 10 (dated from 03.10.2020) germline genomes. Participants who had been recruited with a diagnosis of FH (n=536), as previously described,^5^ were excluded from the analysis. Further methods used to estimate the cohort participants’ relatedness and genetic ancestry are shown in the online supplemental methods.

### WGS data analysis

The WGS methodology and baseline data processing are described in the online supplemental methods. All analyses were performed within the Genomics England Research Environment. Sequencing data for *LDLR*, *APOB*, *PCSK9* and *APOE* genes were extracted from the AggV2 dataset (genes’ coordinates are shown in online supplemental table S1). Variants, with PASS quality (see online supplemental methods), were filtered by gnomAD v4 minor allele frequency (MAF) <0.001 (maximum across the ancestry subgroups), which is higher than the most common single FH-causing variant (the *APOB* p.Arg3527Gln, global gnomAD MAF=0.0004), to remove LB variants. Filtered variants were interpreted as described below.

### SVs analysis

Structural variants (SVs) in the *LDLR* gene are known to account for approximately 10% of all *LDLR* pathogenic variants.^14^ A whole-gene duplication of *PCSK9,* interpreted as a gain-of-function mutation, has been reported^15^; however, it is very rare. SV calls were generated using MANTA.^16^ Putative copy number variations and SVs were intersected with the coordinates of the hg38 genome, using bedtools (v2.19.1). Variants overlapping with the *LDLR* and *PCSK9* gene sequences were analysed.

### Variant interpretation

After the initial variant frequency filtering step, variants in the FH gene regions were reviewed against ClinGen FH Variant Curation Expert Panel (VCEP) classifications. Variants that have not yet been reviewed by ClinGen FH VCEP were analysed using the adapted ACMG criteria^7^ by two independent groups of reviewers (London and Oxford). Variants classified as VUS were additionally reviewed using the recently published high-throughput functional assay data,^17^ to aid the classification. Variants with either LDL-C uptake or LDLR cell-surface abundance scores below 0.5 were given PS3_Moderate criteria. Any individual carrying a variant designated P or LP was given the diagnosis of Heterozygous FH (HeFH), while those carrying designated B/LB/VUS variants were not HeFH. The *APOE* gene was analysed only for the presence of one variant (GRCh38 genomic position chr19:44 908 791 GCTC>G, p.Leu167del), which has been previously shown to cause FH.^18^

### Phenotypic data

100KGP participants’ clinical data records were obtained using the Genomics England Participant Explorer tool within the Research Environment. The data were obtained from the most recent version release, V.19 (31 October 2024). The sources of the clinical data for rare disease participants included NHS inpatients/outpatients hospital information and diagnoses, NHS emergency care and NHS imaging procedures. The data search by clinical concept included searching for hypercholesterolaemia and related terms, using the following code systems: ICD10, HPO, OPCS and SNOMED.

## Results

### 100KGP cohort characteristics

Of the 77 260 non-FH participants included in the AggV2 dataset, 40 998 (53%) were female. The mean (SD) age at recruitment was 41.3 (SD=22) years (median (IQR) = 42 (IQR=31) years). The genetic relatedness analysis identified 54 818 unrelated participants. The majority of genetically unrelated participants in the analysed dataset were recruited as part of the rare disease programme (73%); the remaining were part of the cancer programme.

Using inferred genetic ancestry of unrelated participants, 44 876 (81.9%) individuals were classified as European, 4409 (8%) as South Asian, followed by 1553 (2.8%) classified as African. East Asian and American genetic ancestry was inferred in less than 500 participants, and in the remaining 3399 (6.25%) of participants, genetic ancestry could not be confidently linked to a specific group (called ‘unassigned’).

### FH variant analyses

**Figure 1:**
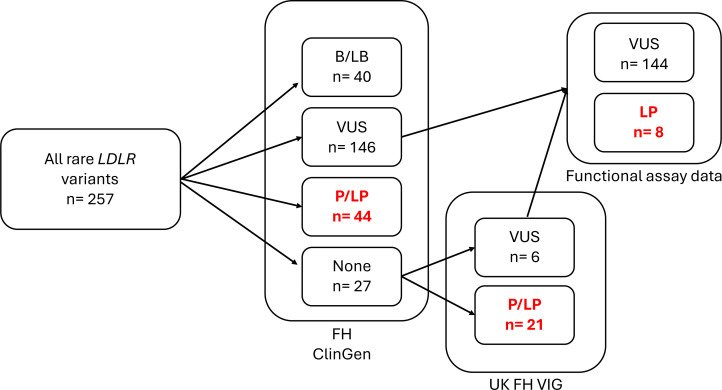
Workflow for the *LDLR* variants interpretation. All rare *LDLR* variants were annotated with ClinGen FH VCEP classification. Variants for which no ClinGen annotation was available were reviewed by two independent reviewer groups, including members of the UK FH Variant Interpretation Group (UK FH VIG). Data from the functional assay study on LDL-C uptake and LDLR cell-surface abundance^17^ was applied to VUSs to aid their interpretation. B, benign; FH, Familial Hypercholesterolaemia; LB, likely benign; LDL-C, low-density lipoprotein-cholesterol; LP, likely pathogenic; P, pathogenic; VCEP, Variant Curation Expert Panel; VUS, variant of uncertain significance.

For *LDLR*, 257 different variants were identified, as shown in figure 1. Out of these, 230 variants have already been reviewed and classified by the expert consortium, the ClinGen FH VCEP. These included 40 B or LB, 146 VUSs and 44 LP and P variants. The remaining 27 variants with no ClinGen classification were then reviewed using the *LDLR*-specific ACMG criteria^7^ by two independent groups of expert reviewers, one based in London and another in Oxford. Of these 27 variants, six were classified as VUSs and 21 were LP or P. In addition, the VUSs were reviewed using the recently published high-quality functional evidence data on the LDL-C uptake and LDLR cell surface abundance.^17^ This step reclassified eight VUSs to the LP variant category. Overall, of the identified *LDLR* variants, after expert curation, 40 (15.6%) were B/LB, 144 (56%) were VUS and 73 (28.4%) were LP/P (figure 1).

For *APOB and PCSK9,* 1516 and 257 different variants were identified respectively, with over 90% having either no ClinVar or ClinGen designation or being classified as B, LB or VUS, and these were not examined further. For *APOB,* any truncation-causing variant would be designated LP/P, but the majority of these cause hypocholesterolaemia, and not FH. For *PCSK9,* any loss-of-function variant would be designated LP/P, but only gain-of-function variants cause FH. Therefore, manual curation and expert opinion was used for all *APOB* and *PCSK9* variants. This resulted in four different *APOB* variants and three different *PCSK9* variants designated as LP/P (online supplemental table S2). No whole-gene *PCSK9* duplications were detected.

### FH variant prevalence

A heterozygous P or LP FH variant was found in 167 of 54 818 unrelated participants (0.3%) giving a cohort prevalence of 1:328 (95% CIs 1:285 to 1:386). No significant difference in FH variant frequency was observed between the main genetic ancestry groups (online supplemental table S3) with European participants having an FH variant in 1:348 (95% CI 1 in 297 to 1 in 420), South Asian 1:276 (95% CI 1 in 170 to 1 in 482) and African 1:388 (1 in 152 to 1 in 1424).

### FH variant spectrum

**Figure 2:**
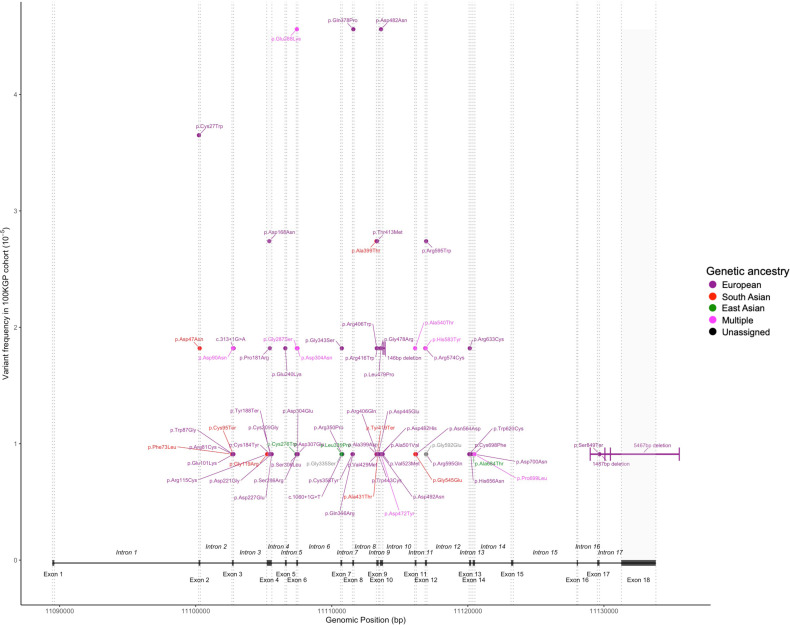
Map of FH variants in the *LDLR* gene by their frequency and genetic ancestry they were found in. 100KGP, 100 000 Genomes Project; FH, Familial Hypercholesterolaemia.

**Figure 3:**
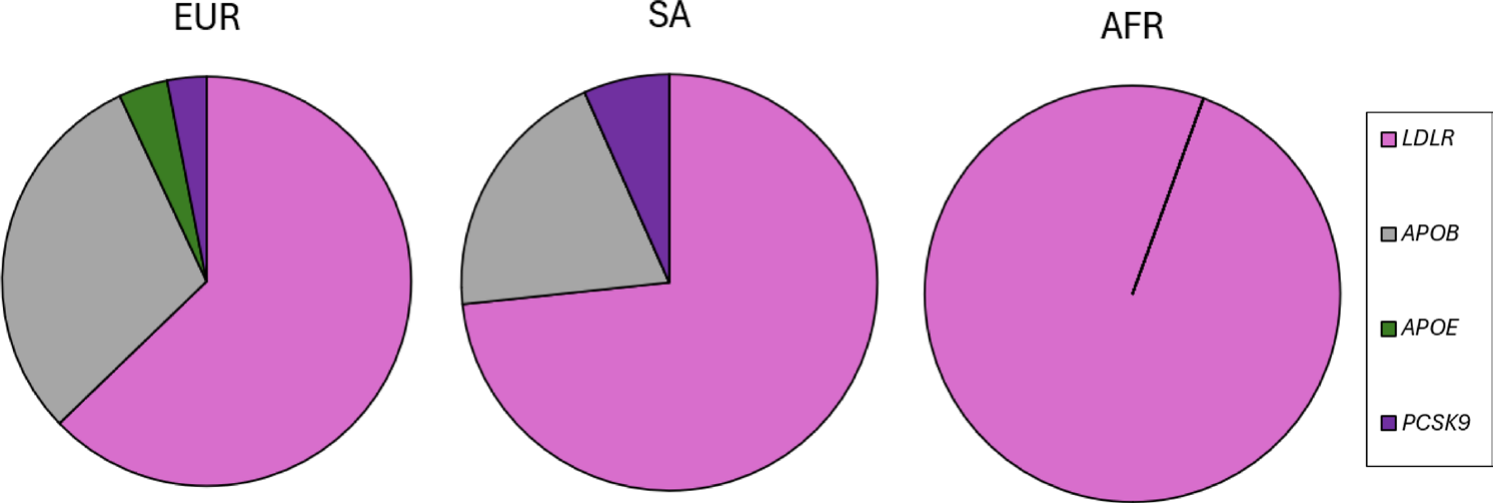
Spectrum of FH variants grouped by gene per three major genetic ancestry groups. AFR, African; EUR, European; FH, Familial Hypercholesterolaemia; SA, South Asian.

In the *LDLR* gene, 74 different FH-causing variants in 113 unrelated subjects were identified, accounting for 67% of all FH causes in this cohort. As shown in online supplemental table S2 and figure 2, variants were found across the entire gene, including splice variants and large gene rearrangements. In the *APOB* gene, four different variants were found in 46 unrelated subjects (online supplemental table S2), with the *APOB* p.Arg3527Gln variant found in 35 unrelated individuals of European ancestry, and the p.Arg3527Trp variant found in three South Asian and one East Asian unrelated participants. For the *PCSK9* gene, three different variants were found in five subjects (3% of all FH causes), and for the *APOE* gene, the p.Leu169del variant was found in six unrelated subjects, accounting for 3.5% of FH variants. As shown in figure 3, the relative proportions of the affected FH genes varied by genetic ancestry, with the *APOE* variant found only in the Europeans, and Africans having FH variants found only in the *LDLR* gene.

Two individuals had two FH-causing variants and would therefore have received the diagnosis of Homozygous FH (HoFH). One female participant of European ancestry, aged 70–80 years, who was part of the rare disease group, was found to have *APOB* p.Arg3527Gln and *LDLR* p.Asn564Asp FH-causing variants. The database had no records of cardiovascular tests/outcomes from hospital data for this individual. The *LDLR* variant was found in two other family members. A second participant was homozygous for *APOB* p.Arg3527Trp. This individual, a singleton, was a male of Indian background, aged 40–50 years, with no electronic health records of CAD.

### Characteristics of individuals with pathogenic FH variants

There were limited data available on the characteristics of the cohort, but of the 167 FH unrelated individuals who had a heterozygous LP/P variant, 55% were female with a mean age at recruitment of 50.2 (SD=16.1) years (median 49 (IQR=26) years). Participants’ medical history was reviewed using linked secondary clinical datasets provided for 100KGP participants. These included Hospital Episodes Statistics from NHS England. The clinical data were searched for ‘pure hypercholesterolaemia’ and its related terms, most of which were contained within the admitted patients care data. Of probands with an FH variant, 67 (40%) had a record of hypercholesterolaemia, compared with the non-variant cohort, where 6193/54 651 (11%) had hypercholesterolaemia (Pearson’s χ2 test p <2.2*10^-16^). There was no difference in the FH variant prevalence between participants who were referred through the rare disease (118 out of 40 011 (0.3%)) and the cancer (46 out of 14 489 (0.32%)) programmes (Pearson’s χ2 test p =0.51).

Of 22 442 genetic relatives, a HeFH-causing variant was found in 77 participants. Of all those with a HeFH variant (including relatives), 43 participants were under the age of 18 years at the time of recruitment to 100KGP, while 90 were over 50 years old. As shown in online supplemental figure S1, the age distribution and mean age at recruitment of all individuals with an FH-causing variant was 41.3 (+21.7) years (median 44 (IQR 28.5) years), which was similar to the to the age distribution in the overall sample.

## Discussion

Using WGS data from 77 260 participants of the 100KGP, we identified overall 169 index cases (including two homozygotes) and 77 relatives with an FH-causing variant in either *LDLR*, *APOB*, *APOE* or *PCSK9*. The overall frequency of HeFH in the index cases of 1:328 (1:285–1:386) was similar to the frequency in the UK Biobank (UKB) cohort of ~150 000 individuals^19^ and to the Million Veteran Program in the USA (~450 000 individuals).^20^ Since untreated individuals with FH have an elevated risk of early death from cardiovascular disease,^4^ this may have resulted in a lower prevalence estimate, although the impact of this is likely to be small, and the mean age of participants in the 100KGP was ~50 years, which is similar to that of the UKB participants.

Using the genetic ancestry information, we observed no significant difference in FH variant frequency between European, African and South Asian participants, replicating our previously reported findings in UKB.^19^ The spectrum of variants in FH genes in Europeans resembled the UKB cohort, with the majority having a disease-causing variant in *LDLR* (67% in 100KGP vs 77% in UKB), followed by *APOB* (26.5% in 100KGP vs 21% in UKB). The proportion of *APOB* variants observed in the non-patient cohorts (100KGP and UKB) is significantly higher than that reported in lipid clinic populations (online supplemental table S4), where *APOB* accounts for approximately 11% of FH cases.^14 21^ This discrepancy may reflect the relatively milder impact of *APOB* variants on LDL-C concentrations. Individuals with an *APOB*-related FH variant are less likely to present with severe hypercholesterolaemia and thus may not meet the threshold for referral to lipid clinics. As a result, such variants could be underrepresented in clinically ascertained cohorts compared with population-based studies. Since pathogenic FH variants increase the risk of CAD regardless of the LDL-C concentrations,^2^ the opportunity to offer treatment and prevention measures in those milder FH cases is likely to be missed.

The *APOB*-related causes of FH were mainly due to the p.Arg3527Gln variant, observed only in Europeans, and another variant at the same amino acid position p.Arg3527Trp, which occurs mainly in individuals of Asian ancestry. The *APOE* p.Leu167del variant was found only in European ancestry group, as we previously observed in the UKB cohort.^19^

The analysis of NHS clinical data, although limited to secondary care records, showed that 40.1% (n=67) of the unrelated participants and 54.3% when including the relatives with FH variant had a record of hypercholesterolaemia, which was significantly higher than the non-HeFH cohort, where 11.3% (n=6193) had hypercholesterolaemia. However, this low proportion of those with an FH variant with recorded hypercholesterolaemia is not unexpected since less than 10% of the predicted number of FH individuals (based on the 1:288 population prevalence)^19^ have yet been identified^8^ with this low diagnosis of FH in general practice leading to undertreatment and high CVD risk.

Our analysis found an FH-causing variant in 43 children (participants who were younger than 18 years at the time of recruitment). Individuals with an FH variant are likely to have high blood concentrations of LDL-C from birth and leading to elevated risk of premature CAD. Therefore, all individuals with a pathogenic FH variant over the age of 10 years should be considered for LLT, and all over the age of 18 years should be taking LLT.^22 23^ Although we do not have data on the proportion of the FH-variant cohort who are on LLT, it is likely that the majority of these subjects at the time of recruitment were unaware of their extremely high risk of premature CAD and were either being offered no LLT or were not being treated with high intensity LLT as is now recommended.^22 23^

Two individuals were found to have two FH-causing variants and would thus have HoFH. HoFH is usually diagnosed in childhood and is often associated with onset of CAD in the second decade of life,^24^ while both of these individuals are in middle age and have no documented evidence of CAD. The *APOB* variant found in these two individuals is known to be associated with lower LDL-C concentrations and with a lower risk of CAD than *LDLR* gene variants,^25^ which is the likely explanation for their unexpected longevity.

One of the aims of the 100KGP was to examine the feasibility and acceptability of returning clinically actionable secondary findings, referred to as AFs, and more than 90% of participants consented to this. Previous mixed-methods research found that 100KGP participants who received an FH AF valued the information, seeing it as an opportunity to be proactive about their health and make changes to diet and exercise.^10^ Professionals involved in returning AFs from the 100KGP were also generally positive about offering clinically actionable AFs, such as FH, within routine NHS clinical care.^26^ For reporting to a clinician and an identified individual, there needs to be a high degree of certainty that the variant is FH-causing. In addition, a higher level of certainty is required for any novel variant finding in a population-based sample than in an individual with the clinical phenotype of FH patient. As discussed above, in individuals of non-European ancestry, the proportion of novel variants identified is higher than in those of European ancestry, but while some of these may actually be FH-causing, because of a lack of data, they have remained as VUS. Since the prevalence of FH-causing variants is similar in all three ancestry groups, the likelihood of finding a reportable AF for FH in people from a range of different backgrounds can be made with confidence. We have also shown that the LDL-C-raising effect of a pathogenic FH variant is the same in three major ancestry groups in the UK,^19^ implying that AFs would be of similar clinical value in all groups. However, reporting AFs needs to be carried out with caution, for example, using culturally specific counselling, since this may require different approaches in individuals of different ancestry to ensure the information is found to be useful to all participants.

One of the limitations of the study is that the cohort was enriched for rare disease and cancer cases, making it possible that it is unrepresentative of the general population with respect to the prevalence of FH cases. However, since the overall prevalence figure (1:328) is not significantly different from that previously reported in non-selected population-based UK samples (such as UKB), this supports the view that this sample does not have a strong ascertainment bias. While we used gnomAD to assess the frequency of identified variants in the non-European ancestry groups (which now contains information on >37 500 individuals of African ancestry and 45 500 of South Asian ancestry), and the ClinGen expertise to assign pathogenicity, we accept that it is likely that a proportion of the novel variants classified as VUS, because of current lack of data, may later be classified as P/LP. While it is not possible to give an accurate estimate of the extent of this effect, it may be that the true prevalence of FH-causing variants in these groups is 5%–10% higher.

A further limitation of the study is that we have not carried out extensive analysis to identify large insertions or deletions in *APOB*, but this is unlikely to have had a significant impact on the overall frequency of FH-causing variants. While large ins/dels have been reported to explain 5–10% of all identified *LDLR* variants in UK FH patients,^14^ the prevalence of such large ins/dels in *APOB* is likely to be much lower.

Another weakness to this study is that there are minimal and incomplete clinical data available for the participants identified with carrying an FH-causing variant, which precluded more detailed analysis such as genotype–phenotype correlation. We are therefore unable to comment on the extent to which these subjects and their referring clinician or general practitioner already knew of their hypercholesterolaemia and CAD risk, and to what extent they were previously, or following their AF, offered and were adhering to lifestyle and LLT. We also do not have any data as to which variants have been designated as P/LP and therefore should be reported as AFs and which have been designated as VUS and so not reported. Finally, no data are available on whether a higher proportion of FH-variant individuals of European ancestry have received AFs compared with those of different groups. Further research to address these issues is warranted.

FH remains a globally highly underdiagnosed^23^ and undertreated^27^ disease. Currently in the UK, the only implemented screening strategy for FH is cascade testing, which requires an FH proband to be identified.^22^ Large-scale population-based genomics projects offer the possibility for an opportunistic screening of FH and would lead to the identification of those with an FH variant earlier in life than in the 100KGP group where the mean age at diagnosis of index cases was 50 years. Although HeFH is not explicitly included in the Genomics England Generation Study,^28^ the 10-Year Health Plan for England^29^ outlines a strong ambition to integrate genomic information into routine preventive care, identifying cardiovascular disease as a key priority. This includes the use of polygenic risk scores and WGS to inform earlier and more personalised interventions. Our data support the practical utility of this approach in the general population regardless of ancestry.

## Supplementary material

10.1136/jmg-2025-111201 online supplemental file 1

## Data Availability

Data may be obtained from a third party and are not publicly available.
