# Supplementary material for: Frequency of familial hypercholesterolaemia-causing genetic variants in the 100 000 Genomes Project cohort: whole genome sequencing analyses of 77 260 participants
Source: J Med Genet. 2026 Feb 10;63(5):e111201. doi: 10.1136/jmg-2025-111201 (PMC13151451; doi:10.1136/jmg-2025-111201)
Supplement: online supplemental file 1 [file jmg-63-5-s001.docx]

**SUPPLEMENTARY MATERIAL**

**Frequency of Familial Hypercholesterolemia-causing genetic variants in the 100,000 Genomes Project cohort: whole genome sequencing analyses of 77,260 participants.**

Marta Futema^1,2^, Martin Bird^1,3^, Ash Haeger^4^, Ellen Pinder^4^, Anthony W O’Rourke^4^, Elijah R Behr^1^, Steve E Humphries^2^

^1^Cardiovascular and Genomics Research Institute, School of Health & Medical Sciences, City St George's, University of London, London, UK.

^2^Institute of Cardiovascular Science, Faculty of Population Health, University College London, London, UK

^3^Clinical Pharmacology and Precision Medicine, William Harvey Research Institute, Queen Mary University of London, Charterhouse Square, London, EC1M 6BQ

^4^Oxford Genetic Laboratories, Oxford University Hospitals NHS Foundation Trust, The Churchill Hospital, Oxford, UK

Corresponding author: Marta Futema mfutema@sgul.ac.uk

**SUPPLEMENTARY METHODS**

**Whole Genome Sequencing**

All samples were sequenced with 150bp paired-end reads using Illumina HiSeq X and uniformly processed on the Illumina North Star Version 4 Whole Genome Sequencing Workflow (NSV4, version 2.6.53.23), which comprises the iSAAC Aligner (version 03.16.02.19) and Starling Small Variant Caller (version 2.4.7). Samples were aligned to the Homo Sapiens NCBI GRCh38 assembly with decoys. Single-sample gVCFs were aggregated using the Illumina software gVCF genotyper (version: 2019.02.26). Variant normalisation and decomposition was implemented by vt (version 0.57721).

An aggregate multi-sample VCF (AggV2) was generated by Genomics England (<https://re-docs.genomicsengland.co.uk/aggv2/>), which comprised variant call data for 78,195 germline genomes aligned to human genome GRCh38. Variants were annotated using the Ensembl Variant Effect Predictor (VEP v99) (36).

**Sample QC**

Samples with contamination>0.03 (estimated using verifyBamID), excess of chimeric reads>5%, median fragment size<250bp, percentage of AT dropout>10%, percentage of mapped reads<60% were excluded from the analysis.

**Variant QC**

Details about the Genomics England WGS data structure and site quality control have been published (35). Definition of the PASS variant quality is shown in the following table:

| Filter | Description |
| --- | --- |
| PASS | PASS value is given if all below listed filters passed |
| missingness | Missingness (fully missing genotypes with DP=0) ≤ 5% |
| depth | Median Depth ≥ 10 |
| GQ | Median GQ ≥ 15 |
| ABratio | Percentage of het calls not showing significant allele imbalance for reads supporting the ref and alt alleles ≥ 25% |
| completeGTRatio | Percentage of complete sites (sites with no missing data) ≥ 50% |
| phwe_eur | mid p-value for deviations from HWE in unrelated samples of inferred European ancestry ≥ 1e^-5^ |

**Genetic ancestry and relatedness definition**

Data generated data by Genomics England, as described on their website: <https://re-docs.genomicsengland.co.uk/ancestry_inference/> and <https://re-docs.genomicsengland.co.uk/principal_components/> was used to infer genetic ancestry of all participants. Briefly, principal components (PCs) and SNP loadings were calculated for 1KGP3 by using GCTA v.1.93.1_beta on a 30K set of high-quality independent variants. Next, the 1KGP3 SNP loadings were used to calculate projected individual loadings for the participants.

To assign ancestry, a random forest classifier was used (rpackage: randomForest) on the 1KGP3 samples using 6 PCs from the 1KGP3 PCs and superpopulation labels (EUR, AFR, AMR, SAS, EAS). The trained model was used on the projected loadings of genomicsengland100kgp participants to calculate probabilities of assignment to each super-population. A probability cut-off threshold T≥ 0.8 was then used to assign each individual to a superpopulation ancestry. 95,829 of high confidence LD-pruned biallelic SNPs to be used on all downstream analyses. The SNPs were selected based on the following criteria:

1. Include autosomal, bi-allelic SNPs only
2. Keep variants which are common (MAF>1%) in both aggV2 and the 1KGP3
3. Missingness < 1%
4. Median GQ ≥ 30
5. Median Depth  ≥ 30
6. AB Ratio ≥  0.9
7. Completeness ≥ 0.9
8. Exclude variants in complex regions, as defined in the ['high LD exclusion regions' file](https://re-docs.genomicsengland.co.uk/aggv2_file_manifest/)
9. Remove all SNPs where the ref/alt combination was AT or GC (A/T, T/A, G/C, C/G), to avoid ambiguous allele swaps
10. LD prune using plink version v1.9 with an r^2^  0.1, 500kb window

Remove all SNPs which are out of Hardy Weinberg Equilibrium (HWE) in any of the afr, eas, eur or sas super-populations, with a p-value cutoff of pHWE < 1e^-5^

Using the high confidence SNPs, Genomics England generated a pairwise kinship matrix using the PLINK2 implementation of the KING_Robust algorithm.

These were then partitioned into related (up to, and including third degree relationships) and unrelated sample lists using the PLINK2 --king-cutoff relationship-pruning algorithm, with a threshold of 0.0442.

**FIGURE S1.**

**Histogram of ages at recruitment in those carrying an FH-causing variant (lower graph) compared to all 100KGP participants (upper graph).**

**
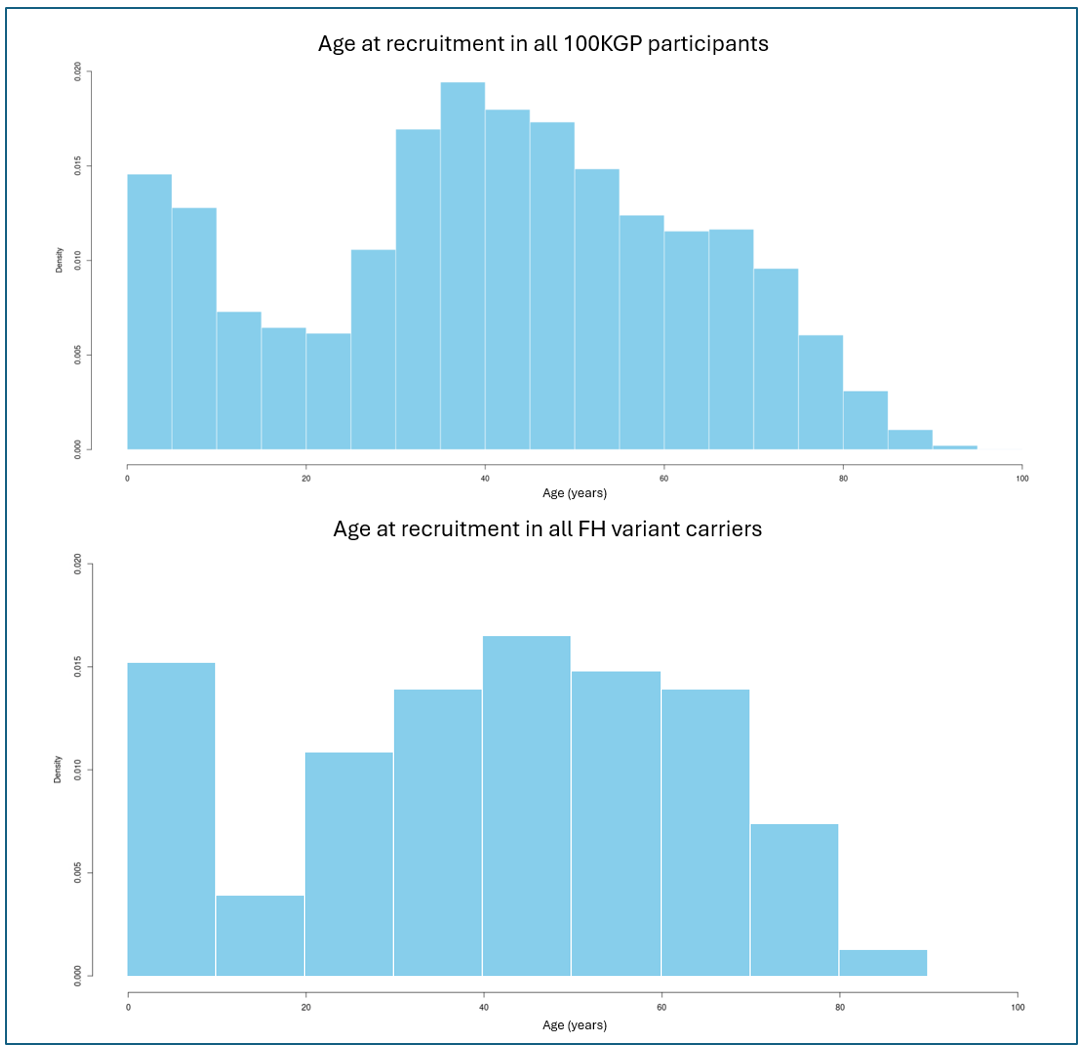
**

**TABLE S1.**

**Genomic coordinates on human genome GRCh38 for FH genes.**

| **Gene name** | **Chromosome number** | **Start coordinate** | **End coordinate** |
| --- | --- | --- | --- |
| *LDLR* | 19 | 11,089,262 | 11,133,820 |
| *APOB* | 2 | 21,001,429 | 21,044,073 |
| *APOE* | 19 | 44,905,791 | 44,909,393 |
| *PCSK9* | 1 | 55,039,347 | 55,064,852 |

**TABLE S2.**

**Pathogenic and likely pathogenic FH variants found in unrelated participants.**

| **FH gene** | **Chromosome** | **Genomic position start** | **cDNA** | **Protein** | **N of probands** | **Genetic ancestry** |
| --- | --- | --- | --- | --- | --- | --- |
| *LDLR* | chr19 | 11100236 | ENST00000558518.6:c.81C>G | ENSP00000454071.1:p.Cys27Trp | <5 | EUR,U |
| *LDLR* | chr19 | 11100294 | ENST00000558518.6:c.139G>A | ENSP00000454071.1:p.Asp47Asn | <5 | SA |
| *LDLR* | chr19 | 11102692 | ENST00000558518.6:c.219C>A | ENSP00000454071.1:p.Phe73Leu | <5 | SA,U |
| *LDLR* | chr19 | 11102714 | ENST00000558518.6:c.241C>T | ENSP00000454071.1:p.Arg81Cys | <5 | EUR |
| *LDLR* | chr19 | 11102732 | ENST00000558518.6:c.259T>G | ENSP00000454071.1:p.Trp87Gly | <5 | EUR |
| *LDLR* | chr19 | 11102741 | ENST00000558518.6:c.268G>A | ENSP00000454071.1:p.Asp90Asn | <5 | EUR,AFR |
| *LDLR* | chr19 | 11102758 | ENST00000558518.6:c.285C>A | ENSP00000454071.1:p.Cys95Ter | <5 | SA |
| *LDLR* | chr19 | 11102774 | ENST00000558518.6:c.301G>A | ENSP00000454071.1:p.Glu101Lys | <5 | EUR |
| *LDLR* | chr19 | 11102787 | ENST00000558518.6:c.313+1G>A | - | <5 | EUR |
| *LDLR* | chr19 | 11105249 | ENST00000558518.6:c.343C>T | ENSP00000454071.1:p.Arg115Cys | <5 | EUR |
| *LDLR* | chr19 | 11105261 | ENST00000558518.6:c.355G>A | ENSP00000454071.1:p.Gly119Arg | <5 | SA |
| *LDLR* | chr19 | 11105408 | ENST00000558518.6:c.502G>A | ENSP00000454071.1:p.Asp168Asn | <5 | EUR |
| *LDLR* | chr19 | 11105448 | ENST00000558518.6:c.542C>G | ENSP00000454071.1:p.Pro181Arg | <5 | EUR |
| *LDLR* | chr19 | 11105457 | ENST00000558518.6:c.551G>A | ENSP00000454071.1:p.Cys184Tyr | <5 | EUR |
| *LDLR* | chr19 | 11105470 | ENST00000558518.6:c.564C>G | ENSP00000454071.1:p.Tyr188Ter | <5 | EUR |
| *LDLR* | chr19 | 11105531 | ENST00000558518.6:c.625T>G | ENSP00000454071.1:p.Cys209Gly | <5 | EUR |
| *LDLR* | chr19 | 11105568 | ENST00000558518.6:c.662A>G | ENSP00000454071.1:p.Asp221Gly | <5 | EUR |
| *LDLR* | chr19 | 11105587 | ENST00000558518.6:c.681C>G | ENSP00000454071.1:p.Asp227Glu | <5 | EUR |
| *LDLR* | chr19 | 11106588 | ENST00000558518.6:c.718G>A | ENSP00000454071.1:p.Glu240Lys | <5 | EUR |
| *LDLR* | chr19 | 11107402 | ENST00000558518.6:c.828C>G | ENSP00000454071.1:p.Cys276Trp | <5 | EA |
| *LDLR* | chr19 | 11107432 | ENST00000558518.6:c.858C>A | ENSP00000454071.1:p.Ser286Arg | <5 | EUR |
| *LDLR* | chr19 | 11107433 | ENST00000558518.6:c.859G>A | ENSP00000454071.1:p.Gly287Ser | <5 | EUR,AFR |
| *LDLR* | chr19 | 11107436 | ENST00000558518.6:c.862G>A | ENSP00000454071.1:p.Glu288Lys | 5 | EUR,AFR,U |
| *LDLR* | chr19 | 11107484 | ENST00000558518.6:c.910G>A | ENSP00000454071.1:p.Asp304Asn | <5 | EUR,AFR |
| *LDLR* | chr19 | 11107486 | ENST00000558518.6:c.912C>G | ENSP00000454071.1:p.Asp304Glu | <5 | EUR |
| *LDLR* | chr19 | 11107491 | ENST00000558518.6:c.917C>T | ENSP00000454071.1:p.Ser306Leu | <5 | EUR |
| *LDLR* | chr19 | 11107495 | ENST00000558518.6:c.921T>G | ENSP00000454071.1:p.Asp307Glu | <5 | EUR |
| *LDLR* | chr19 | 11110714 | ENST00000558518.6:c.1003G>A | ENSP00000454071.1:p.Gly335Ser | <5 | U |
| *LDLR* | chr19 | 11110727 | ENST00000558518.6:c.1016T>C | ENSP00000454071.1:p.Leu339Pro | <5 | EA |
| *LDLR* | chr19 | 11110738 | ENST00000558518.6:c.1027G>A | ENSP00000454071.1:p.Gly343Ser | <5 | EUR |
| *LDLR* | chr19 | 11110760 | ENST00000558518.6:c.1049G>C | ENSP00000454071.1:p.Arg350Pro | <5 | EUR |
| *LDLR* | chr19 | 11110772 | ENST00000558518.6:c.1060+1G>T | - | <5 | EUR |
| *LDLR* | chr19 | 11111526 | ENST00000558518.6:c.1073G>A | ENSP00000454071.1:p.Cys358Tyr | <5 | EUR |
| *LDLR* | chr19 | 11111550 | ENST00000558518.6:c.1097A>G | ENSP00000454071.1:p.Gln366Arg | <5 | EUR |
| *LDLR* | chr19 | 11111586 | ENST00000558518.6:c.1133A>C | ENSP00000454071.1:p.Gln378Pro | 5 | EUR |
| *LDLR* | chr19 | 11113286 | ENST00000558518.6:c.1195G>A | ENSP00000454071.1:p.Ala399Thr | <5 | SA,U |
| *LDLR* | chr19 | 11113287 | ENST00000558518.6:c.1196C>A | ENSP00000454071.1:p.Ala399Asp | <5 | EUR |
| *LDLR* | chr19 | 11113307 | ENST00000558518.6:c.1216C>T | ENSP00000454071.1:p.Arg406Trp | <5 | EUR,U |
| *LDLR* | chr19 | 11113308 | ENST00000558518.6:c.1217G>A | ENSP00000454071.1:p.Arg406Gln | <5 | EUR |
| *LDLR* | chr19 | 11113329 | ENST00000558518.6:c.1238C>T | ENSP00000454071.1:p.Thr413Met | <5 | EUR |
| *LDLR* | chr19 | 11113337 | ENST00000558518.6:c.1246C>T | ENSP00000454071.1:p.Arg416Trp | <5 | EUR |
| *LDLR* | chr19 | 11113348 | ENST00000558518.6:c.1257C>G | ENSP00000454071.1:p.Tyr419Ter | <5 | SA |
| *LDLR* | chr19 | 11113376 | ENST00000558518.6:c.1285G>A | ENSP00000454071.1:p.Val429Met | <5 | EUR |
| *LDLR* | chr19 | 11113382 | ENST00000558518.6:c.1291G>A | ENSP00000454071.1:p.Ala431Thr | <5 | SA |
| *LDLR* | chr19 | 11113420 | ENST00000558518.6:c.1329G>C | ENSP00000454071.1:p.Trp443Cys | <5 | EUR |
| *LDLR* | chr19 | 11113426 | ENST00000558518.6:c.1335C>G | ENSP00000454071.1:p.Asp445Glu | <5 | EUR |
| *LDLR* | chr19 | 11113590 | ENST00000558518.6:c.1414G>T | ENSP00000454071.1:p.Asp472Tyr | <5 | EUR,SA |
| *LDLR* | chr19 | 11113608 | ENST00000558518.6:c.1432G>A | ENSP00000454071.1:p.Gly478Arg | <5 | EUR,U |
| *LDLR* | chr19 | 11113612 | ENST00000558518.6:c.1436T>C | ENSP00000454071.1:p.Leu479Pro | <5 | EUR |
| *LDLR* | chr19 | 11113620 | ENST00000558518.6:c.1444G>C | ENSP00000454071.1:p.Asp482His | <5 | EUR |
| *LDLR* | chr19 | 11113620 | ENST00000558518.6:c.1444G>A | ENSP00000454071.1:p.Asp482Asn | 5 | EUR,U |
| *LDLR* | chr19 | 11113650 | ENST00000558518.6:c.1474G>A | ENSP00000454071.1:p.Asp492Asn | <5 | EUR |
| *LDLR* | chr19 | 11113678 | ENST00000558518.6:c.1502C>T | ENSP00000454071.1:p.Ala501Val | <5 | EUR |
| *LDLR* | chr19 | 11113743 | ENST00000558518.6:c.1567G>A | ENSP00000454071.1:p.Val523Met | <5 | EUR |
| *LDLR* | chr19 | 11116125 | ENST00000558518.6:c.1618G>A | ENSP00000454071.1:p.Ala540Thr | <5 | EUR,SA |
| *LDLR* | chr19 | 11116141 | ENST00000558518.6:c.1634G>A | ENSP00000454071.1:p.Gly545Glu | <5 | SA |
| *LDLR* | chr19 | 11116197 | ENST00000558518.6:c.1690A>G | ENSP00000454071.1:p.Asn564Asp | <5 | EUR,U |
| *LDLR* | chr19 | 11116873 | ENST00000558518.6:c.1720C>T | ENSP00000454071.1:p.Arg574Cys | <5 | EUR,U |
| *LDLR* | chr19 | 11116900 | ENST00000558518.6:c.1747C>T | ENSP00000454071.1:p.His583Tyr | <5 | SA,EA,U |
| *LDLR* | chr19 | 11116928 | ENST00000558518.6:c.1775G>A | ENSP00000454071.1:p.Gly592Glu | <5 | U |
| *LDLR* | chr19 | 11116936 | ENST00000558518.6:c.1783C>T | ENSP00000454071.1:p.Arg595Trp | <5 | EUR,U |
| *LDLR* | chr19 | 11116937 | ENST00000558518.6:c.1784G>A | ENSP00000454071.1:p.Arg595Gln | <5 | EUR |
| *LDLR* | chr19 | 11120106 | ENST00000558518.6:c.1860G>T | ENSP00000454071.1:p.Trp620Cys | <5 | EUR |
| *LDLR* | chr19 | 11120143 | ENST00000558518.6:c.1897C>T | ENSP00000454071.1:p.Arg633Cys | <5 | EUR |
| *LDLR* | chr19 | 11120212 | ENST00000558518.6:c.1966C>A | ENSP00000454071.1:p.His656Asn | <5 | EUR |
| *LDLR* | chr19 | 11120408 | ENST00000558518.6:c.2026G>A | ENSP00000454071.1:p.Gly676Ser | <5 | EUR |
| *LDLR* | chr19 | 11120432 | ENST00000558518.6:c.2050G>A | ENSP00000454071.1:p.Ala684Thr | <5 | EA,U |
| *LDLR* | chr19 | 11120475 | ENST00000558518.6:c.2093G>T | ENSP00000454071.1:p.Cys698Phe | <5 | EUR |
| *LDLR* | chr19 | 11120478 | ENST00000558518.6:c.2096C>T | ENSP00000454071.1:p.Pro699Leu | <5 | EUR,AFR,U |
| *LDLR* | chr19 | 11120480 | ENST00000558518.6:c.2098G>A | ENSP00000454071.1:p.Asp700Asn | <5 | EUR |
| *LDLR* | chr19 | 11129669 | ENST00000558518.6:c.2546C>A | ENSP00000454071.1:p.Ser849Ter | <5 | EUR |
| *LDLR*_SV | chr19 | 11113770 | 146bp deletion (part of exon 10) | - | <5 | EUR,U |
| *LDLR*_SV | chr19 | 11128992 | 1487bp deletion (intron 16 to intron 17) | - | <5 | EUR |
| *LDLR*_SV | chr19 | 11130072 | 5467bp deletion (from intron 17 to 3’UTR) | - | <5 | EUR |
| *APOB* | chr2 | 21002180 | ENST00000233242.5:c.13242del | ENSP00000233242.1:p.Leu4415Ter | <5 | EUR |
| *APOB* | chr2 | 21006288 | ENST00000233242.5:c.10580G>A | ENSP00000233242.1:p.Arg3527Gln | 35 | EUR |
| *APOB* | chr2 | 21006289 | ENST00000233242.5:c.10579C>T | ENSP00000233242.1:p.Arg3527Trp | <5 | EUR,SA,EA |
| *APOB* | chr2 | 21002393 | ENST00000233242.5:c.13028_13029del | ENSP00000233242.1:p.Tyr4343CysfsTer3 | <5 | EUR,EA |
| *PCSK9* | chr1 | 55039931 | ENST00000302118.5:c.94G>A | ENSP00000303208.5:p.Glu32Lys | <5 | SA |
| *PCSK9* | chr1 | 55052343 | ENST00000302118.5:c.589G>A | ENSP00000303208.5:p.Glu197Lys | <5 | EUR |
| *PCSK9* | chr1 | 55058640 | ENST00000302118.5:c.1496G>A | ENSP00000303208.5:p.Arg499His | <5 | EUR,U |
| *APOE* | chr19 | 44908791 | ENST00000252486.9:c.500_502del | ENSP00000252486.3:p.Leu167del | 6 | EUR,U |

Note. GEL reporting guidelines require variant data in groups with fewer than 5 individuals to be shown as “<5” to prevent participant identification

**TABLE S3.**

**Prevalence of FH variants by genetic ancestry.** Data from genetically unrelated participants. The remaining 21 FH variants were found in either Unassigned genetic ancestry or in East Asian or American participants, excluded from the comparison due to very small sample size. EUR= European, AFR= African, SA= South Asian, CI= confidence intervals

| **Genetic ancestry** | **Total N participants** | **FH variant carriers** | **FH frequency** | **FH prevalence (95%CI)** |
| --- | --- | --- | --- | --- |
| EUR | 44876 | 129 | 0.0029 | 1:348 (1 in 297 to 1 in 420) |
| AFR | 1553 | 4 | 0.0026 | 1:388 (1 in 152 to 1 in 1,424) |
| SA | 4409 | 16 | 0.0036 | 1:276 (1 in 170 to 1 in 482) |

**TABLE S4.**

**Number of probands with FH-causing variants identified in the *APOB* gene *versus* in other FH genes in the current study and as reported in UK Biobank and two clinical FH studies.**

| **Study (reference)** | ***APOB*/Other**  **Number (%age *APOB*)** | **P value vs Clinical FH** |
| --- | --- | --- |
| Current study | 46/123 (26.5%) | P= 2.1x10^-5^ |
| UK BioBank (14) | 105/414 (20.2%) | P = 0.001 |
| Clinical FH (15,16) | 39/294 (11.7%) |  |

Note: in the two clinical FH studies, WGS was not used, so while all individuals with the *APOB* p.Arg3527Gln variant were found some *LDLR/PCSK9* variants were likely to have been missed (and the *APOE* p.Leu169del variant was not included). The consequence of this would be a marginal overrepresentation of the true proportion of *APOB* variants in the clinical FH cohorts.

14 Gratton J, Humphries SE, Futema M. Prevalence of FH-Causing Variants and Impact on LDL-C Concentration in European, South Asian, and African Ancestry Groups of the UK Biobank - Brief Report. Arterioscler Thromb Vasc Biol. 2023;43(9).

15 Taylor A, Wang D, Patel K, Whittall R, Wood G, Farrer M, et al. Mutation detection rate and spectrum in familial hypercholesterolaemia patients in the UK pilot cascade project. Clin Genet. 2010 Jun;77(6):572–80.

16. Futema M, Whittall RA, Kiley A, Steel LK, Cooper JA, Badmus E, et al. Analysis of the frequency and spectrum of mutations recognised to cause familial hypercholesterolaemia in routine clinical practice in a UK specialist hospital lipid clinic. Atherosclerosis. 2013 Jul;229(1):161–8.
